# Accountable survival contrast-learning for optimal dynamic treatment regimes

**DOI:** 10.1038/s41598-023-29106-w

**Published:** 2023-02-08

**Authors:** Taehwa Choi, Hyunjun Lee, Sangbum Choi

**Affiliations:** 1 Department of Biostatistics and Bioinformatics, Duke University, Durham, NC USA; 2 SK Inc. C &C, Seoul, South Korea; 3 Department of Statistics, Korea University, Seoul, South Korea

**Keywords:** Applied mathematics, Statistics

## Abstract

Dynamic treatment regime (DTR) is an emerging paradigm in recent medical studies, which searches a series of decision rules to assign optimal treatments to each patient by taking into account individual features such as genetic, environmental, and social factors. Although there is a large and growing literature on statistical methods to estimate optimal treatment regimes, most methodologies focused on complete data. In this article, we propose an accountable contrast-learning algorithm for optimal dynamic treatment regime with survival endpoints. Our estimating procedure is originated from a doubly-robust weighted classification scheme, which is a model-based contrast-learning method that directly characterizes the interaction terms between predictors and treatments without main effects. To reflect the censorship, we adopt the pseudo-value approach that replaces survival quantities with pseudo-observations for the time-to-event outcome. Unlike many existing approaches, mostly based on complicated outcome regression modeling or inverse-probability weighting schemes, the pseudo-value approach greatly simplifies the estimating procedure for optimal treatment regime by allowing investigators to conveniently apply standard machine learning techniques to censored survival data without losing much efficiency. We further explore a SCAD-penalization to find informative clinical variables and modified algorithms to handle multiple treatment options by searching upper and lower bounds of the objective function. We demonstrate the utility of our proposal via extensive simulations and application to AIDS data.

## Introduction

Dynamic treatment regime (DTR) is an emerging paradigm for maximizing treatment efficacy by providing tailored medicine to each patient^1,2^. Many chronic diseases, such as cancer, human immunodeficiency virus (HIV), and depression, are hard to be cured by a single treatment, requiring continuous disease management. Because human’s clinical information can change over time, sequentially adjusted treatments should be provided in practice, not only based on patients’ clinical history, but also their prior treatment information and intermediate responses. Due to the heterogeneity of the treatment effect affected by the patient’s baseline characteristics, a treatment regime can be defined as a decision rule that assigns a treatment to a patient by taking into account individual features such as genetic, environmental, and social factors. The optimal treatment regime is usually defined as the one that maximizes the average clinical benefit in the potential population for a single treatment. Then a DTR consists of a sequence of optimal treatment regimes, one per stage of intervention, that dictate how to individualize treatments to patients based on evolving treatment and covariate history.

There is a large and growing literature on statistical methods for effectively estimating optimal treatment regimes under multi-stage randomization clinical trials. Since the seminal work by Murphy^1^, numerous methods have been developed to explore personal characteristics such as genetic information or clinical information to find effective data-driven treatment rules. One of statistical approaches for finding optimal treatment regimes is to use a model-based method to evaluate the treatment regimes by positing appropriate statistical models for outcome on predictors, treatment, and predictor-by-treatment interaction, where the interaction term is mainly used to determine the optimal decision rules. Many early works use Q-learning or inverse-probability weighting schemes in single-stage^3–5^ and multi-stage treatment^6–8^ settings. However, as the accessibility of individual information, such as molecular, environmental, and genomic data, increases, these approaches may exhibit a curse of dimensionality and suffer from low accuracy due to potential model mis-specification.

Alternatives to these model-based methods include the outcome-weighted learning (OWL) algorithm and its doubly-robust (DR) versions^9–14^, which directly work on the predictor-by-treatment interaction term by recasting the original search problem for the optimal treatment rule as a problem of minimizing the weighted misclassification error. There, the original 0–1 loss may be substituted by a convex surrogate loss like the hinge loss function to apply a weighted support vector machine (SVM) algorithm for the weighted classification problem. Instead, Zhang and Zhang^13^ directly minimized the non-smooth weighted misclassification error via a generic search algorithm. Tao and Wang^12^ studied the problem of searching optimal treatment rules when there are multiple treatment options. More recent developments explored various modern machine learning techniques, such as Markov decision process or graphical modeling^15–17^, and instrumental variable approaches to deal with possible confounders under observational studies^18,19^. See also Tsiatis et al.^20^ for a comprehensive review of the problem setting for DTR and related statistical methodologies.

In the survival analysis literature, many methods have also been developed to establish optimal treatment rules for survival outcomes, mostly based on outcome regression modeling^8,21,22^ or inverse-probability censoring weighted (IPCW) schemes^23–25^. However, many existing DTR methods for censored data are notoriously complicated, as they often intend to directly maximize nonparametric Kaplan–Meier curves. For example, Jiang et al.^24^ and Zhou et al.^25^ aimed to optimize IPCW-adjusted nonparametric *t*-year survival and cumulative incidence function for a competing risk, respectively, under the counterfactual framework^26^. Their methods are in general computationally unstable, because these nonparametric survival curves are often non-smooth, and thus its estimation may require extra smoothing procedures. Moreover, their algorithms are computationally expensive, because they involve iterative numerical evaluations of the target survival function in each optimization, and also they may not accommodate high-dimensional survival data. Other IPCW-based methods^23,27^ used double inverse-weighting schemes to facilitate censoring and treatment allocation from the classification perspective. Several authors assumed a semiparametric linear regression model and directly calculated the counterfactual survival time through the IPCW adjustment for censored data^8,21^. Although IPCW-based estimation is a convenient and standard way of handling censored data, it is usually sensitive to the amount and distribution of censored variables and is statistically and computationally inefficient even with doubly robust adjustments.

In this article, we propose an accountable contrast-learning algorithm for optimal dynamic treatment regimes with survival endpoints. Our estimating procedure is originated from a doubly-robust weighted classification scheme, which is a model-based contrast-learning method that directly characterizes the interaction terms between predictors and treatments without working on main effects. To reflect the censorship, we adopt the pseudo-value approach^28,29^ that replaces survival quantities with their pseudo-observations for the time-to-event outcome. Unlike many existing approaches, mostly based on outcome regression modeling or IPCW schemes, the pseudo-value methods enable investigators to conveniently apply standard machine learning techniques to censored data with minimal loss of statistical efficiency. We show that pseudo values, designed to handle censoring, can be a natural unbiased substitute for estimating survival quantities when derived from a consistent estimator. Pseudo values are easy to compute and can also be applied to more complex censoring schemes, such as competing risks, restricted mean lifetime, and interval-censoring, etc. Once the pseudo survival responses are obtained, our estimating procedure is based on a penalized survival contrast-learning (PSCL) algorithm to estimate patient-level tailored treatment rules.

The proposed pseudo-value approach for adaptive treatment allocation exhibits two levels of robustness. The first level of robustness is achieved because the proposed method imposes model assumptions only on the predictor-by-treatment interaction term, not on the main-effect term. The other is attained as the form of the contrasting treatment effects is allowed to be doubly-robust by adopting a standard method for complete data. As a result, the proposed learning algorithm is more robust to model mis-specifications, and nonparametric learning methods such as SVM, random forests and boosting can be naturally applied to identify optimal treatment rules. Empirical results on synthetic and real-world datasets show that our proposed methods can achieve superior results under various censoring settings, compared to other competitors.

## Pseudo observations for survival outcomes

We begin by briefly overviewing the pseudo-value approach for survival data^28,29^. Suppose there are *n* random samples. Let $\theta = E[s(T)]$ be a parameter of interest, where $s(\cdot )$ is a measurable function of survival time *T*. For example, one might consider $I(T\ge t)$ and $\min (T,\tau )$ for *s*(*T*), respectively, corresponding to *t*-year survival and restricted mean lifetime up to time $\tau >0$. Pseudo-observations are basically jackknife-type resampling substitutes for unknown survival quantities. To be specific, the pseudo-observation for the *i*th subject can be defined as ${\hat{\theta _i}} = n{\hat{\theta }} - (n-1){\hat{\theta }}^{-i}$, where ${\hat{\theta }}$ is an unbiased estimator of $\theta$ and ${\hat{\theta }}^{-i}$ is the leave-one-out (i.e., jackknife) estimator, based on $n-1$ samples excluding the *i*th object. Note that the pseudo-observation ${\hat{\theta _i}}$ is unbiased estimator, since $E({\hat{\theta _i}}) = nE({\hat{\theta }}) - (n-1)E({\hat{\theta }}^{-i}) = n\theta - (n-1)\theta = \theta .$ This property can be equivalently applied to the survival quantities. For example, the *t*-year survival, $S(t)=P(T\ge t)$, can be approximated by

1 $$\begin{aligned} {{\hat{S}}}_i(t) = n{{\hat{S}}}(t) - (n-1){{\hat{S}}}^{-i}(t), \end{aligned}$$

where ${{\hat{S}}}(t)$ and ${{\hat{S}}}^{-i}(t)$ are nonparametric Kaplan–Meier estimators, based on all *n* samples and $n-1$ samples without the *i*th observation, respectively. Similar techniques can be used to approximate restricted mean lifetime or cumulative incidence rate for a competing risk. In this article, we also focus on the competing risks setting as it includes the standard survival problem as a special case. For the *i*th subject, let $T_i$ and $C_i$ be failure and censoring time variables, respectively, and ${{\textbf{x}}}_i$ be the baseline covariate. Also, let $D_i\in \{1,\ldots ,M\}$ denote the indicator for cause of failure, where *M* is a known number of distinct failure causes, In the presence of censoring, we can actually observe $\{({\tilde{T}}_i,\Delta _i,{{\textbf{x}}}_i),i=1,\ldots ,n\}$, where ${\tilde{T}}_i=\min (T_i,C_i)$ and $\Delta _i=I(T_i\le C_i)D_i$. When the event of interest is the first cause of failure, the primary interest is often the *t*-year cumulative incidence function (CIF), defined as $F_1(t)=P(T_i\le t, D_i=1)$, for which $F_1(t)=E[s_t(T)]$ and $s_t(T)=I(T\le t,D=1)$. This can also be approximated by the pseudo-value approach through the equation

2 $$\begin{aligned} {{\hat{F}}}_{1i}(t)=\int _0^t {{\hat{S}}}_i(s)d{\hat{\Lambda }}_{1i}(s), \end{aligned}$$

where ${\hat{\Lambda }}_{1i}(t)$ is the estimated cause-1 specific cumulative hazard function. Our objective is then to construct an efficient and interpretable DTR rule by minimizing the *t*-year CIF on average. The pseudo-observations can also be computed using functions in the *R: pseudo* package.

A drawback of this basic pseudo-value approach is that it requires a stringent independent assumption between $T_i$ and $C_i$. To relax it to the conditional independent assumption, i.e., $T_i\perp \!\!\!\! \perp C_i|{{\textbf{x}}}_i$, several IPCW-adjusted nonparametric estimators for survival function^30,31^, some of which are available in the *R: eventglm* package^32^, have been developed. For example, one may use the following equations to compute the survival curves under covariate-dependent censoring

3 $$\begin{aligned} {{\hat{S}}}(t) = \frac{\sum _{i=1}^n I(T_i>t){{\hat{v}}}_i}{\sum _{i=1}^n {{\hat{v}}}_i}\quad \text{or}\quad {{\hat{S}}}(t) = n^{-1}\sum _{i=1}^n \frac{I(T_i>t, C_i\ge T_i\wedge t)}{{{\hat{G}}}({\tilde{T}}_i\wedge t |{{\textbf{x}}}_i) }, \end{aligned}$$

where ${{\hat{v}}}_i = I(C_i\ge T_i\wedge t)/{{\hat{G}}}({\tilde{T}}_i\wedge t |{{\textbf{x}}}_i)$. Here, ${{\hat{G}}}({\tilde{T}}_i\wedge t |{{\textbf{x}}}_i)$ is a consistent estimator of $G({\tilde{T}}_i\wedge t |{{\textbf{x}}}_i)=P(C_i > {\tilde{T}}_i\wedge t|{{\textbf{x}}}_i)$, which may be estimated by Cox’s proportional hazards model. Our experience is that two estimators perform similarly and they do not considerably outperform the basic pseudo-value estimator under the strict independent assumption.

## Methods

### Notation and assumptions

Suppose now that patients are treated sequentially with multi-stage treatments. With a slight abuse of notation, we redefine random variables in the following to describe longitudinal trajectories of *K*-stage clinical interventions. Let individuals be identified with $i = 1,\ldots ,n$ and stages be denoted by $k = 1,\ldots ,K$. Let $A_k = a_k\in {\mathscr{A}}_k = \{0,1\}$ and ${{\textbf{x}}}_k$ be the treatment option and covariates, respectively, both observed at the beginning of stage *k*, and let $R_k$ be the reward, such as survival time, when the *k*th treatment $A_k$ is given. Usually, larger reward values are preferable, but smaller values are preferred when CIF is the target objective. Let $\eta _{k}$ be a random indicator that takes value 1 if a patient is alive at the beginning of the *k*th stage and 0 otherwise. By convention, we let $\eta _1=1$ since all recruited patients are at least alive at the first treatment stage. Then, we let ${{\textbf{H}}}_1=\{\eta _1,{{\textbf{x}}}_1\}$ and ${{\textbf{H}}}_k=\{\eta _1,{{\textbf{x}}}_1,A_1,R_1,\ldots ,\eta _{k-1},{{\textbf{x}}}_{k-1},A_{k-1},R_{k-1},\eta _k,{{\textbf{x}}}_k\}$ $(k\ge 2)$ to denote the clinical histories of an individual up to stage *k*. Note that $\{{{\textbf{x}}}_k,A_k,R_k\}$ may be missing data when $\eta _k=0$. By observing all set of rewards, we can then define the overall outcome of interest as $T = m(\eta _1R_1,\ldots ,\eta _KR_K)$, where $m(\cdot )$ is a prespecified function, for example, $T=\sum _{k=1}^K \eta _kR_k$. In the presence of censoring, however, the reward and consequently total reward *T* may not be fully observed. When the components in *T* are censored, we can substitute the target measure $\theta =E[s(T)]$ with the corresponding pseudo-observation ${\hat{\theta _i}}$ for patient *i*. Since the pseudo-value ${\hat{\theta _i}}$ is also a random variable, we shall use the notation *Y* in the following to denote the pseudo-observation of $\theta$.

Now we define the potential outcomes as $T^*({{{\textbf{a}}}}_K)=\sum _{k=1}^K \eta _k R^*_k({{{\textbf{a}}}}_k)$ and correspondingly $Y^*({{{\textbf{a}}}}_K)$, where $R_k^*({{{\textbf{a}}}}_k)$ denotes the potential reward for stage *k* if, possibly contrary to the fact, a patient were given treatments ${{{\textbf{a}}}}_k = (a_1,\ldots ,a_k)\in \{0,1\}^k$. The optimal DTR will then maximize the expectation of the potential reward outcome as each patient were given the best treatment options at all stages. Let $g_k \equiv g_k({{\textbf{H}}}_k)\in \{0,1\}, ~(k=1,\ldots ,K)$ be the treatment regime at the *k*th stage, mapping from the clinical history ${{\textbf{H}}}_k$ to the treatment variable $A_k$. A DTR, observed at the end-of-stage, is defined as ${{{\textbf{g}}}}= (g_1,\ldots ,g_K) \in {\mathscr{G}}$, where ${\mathscr{G}}$ denotes all possible set of treatment regimes. The optimal DTR, denoted by ${{{\textbf{g}}}}^\text{opt}=(g_1^\text{opt},\ldots ,g_{K}^\text{opt})$, is expected to achieve $E[Y^*({{{\textbf{g}}}}^\text{opt})]\ge E[Y^*({{{\textbf{g}}}})]$ for any ${{{\textbf{g}}}}\in {\mathscr{G}}$. We make the following standard assumptions for causal inference to link potential outcomes to observed data^10,33^: (i) *Consistency*, (ii) *Sequential randomization*, and (iii) *Coarsening at random*. Assumption (i) states that the potential outcome coincides with the observed one when a subject is actually given the treatment. Assumption (ii) states that the treatment variable at each stage does not rely on future covariates and treatment history, i.e., $\{\sum _{j\ge l}^K \eta _j R_j^*(a_j):l=k,\ldots ,K \} \perp \!\!\!\! \perp A_k|{{\textbf{H}}}_{k}$. Lastly, assumption (iii) assumes that at the beginning of each stage, the probability of censoring onward is independent of future outcomes, given accrued information. This means that the censoring indicator is conditionally independent of future rewards, i.e., $\{\sum _{j>l}^K \eta _j R_j^*(a_j):l=k,\ldots ,K \} \perp \!\!\!\! \perp \Delta |{{\textbf{H}}}_k$.

### Individualized treatment regimes

To motivate our method, we first consider the simplest single-stage problem (i.e., $K=1$). By convention, it is assumed that the optimal treatment regime $g^{\text{opt}}\in {\mathscr{G}}$ should also satisfy $E\{Y^*(g^{\text{opt}})\} \ge E\{Y^*(g)\}$ for all $g\in {\mathscr{G}}$. By the consistency assumption, the potential pseudo outcome of an arbitrary regime *g* can be linked to observed data as $Y^*(g) = Y^*(1) I\{ g({{\textbf{H}}}) = 1 \} + Y^*(0) I\{ g({{\textbf{H}}}) = 0 \}.$ By letting $\mu _a({{\textbf{H}}}) = E(Y|A=a,{{\textbf{H}}})$, $E\{Y^*(g)\} = E_{{{\textbf{H}}}} [ \mu _1({{\textbf{H}}}) I\{ g({{\textbf{H}}}) = 1 \} +\mu _0({{\textbf{H}}}) I\{ g({{\textbf{H}}}) = 0 \} ]$, where $E_{{{\textbf{H}}}}$ is an expectation with respect to clinical information ${{\textbf{H}}}$. From the classification perspective for decision-making problems^34^, the optimal treatment regime $g^{\text{opt}}$ can be obtained by

4 $$\begin{aligned} g^{\text{opt}}({{\textbf{H}}})= \underset{{g\in {\mathscr{G}}}}{{{\,{\hbox{arg min}}\,}}}\,E_{{\textbf{H}}}\left[ |C({{\textbf{H}}})|\{ I[C({{\textbf{H}}})>0] \ne g({{\textbf{H}}}) \} \right] , \end{aligned}$$

where $C({{\textbf{H}}}) = \mu _1({{\textbf{H}}})-\mu _0({{\textbf{H}}})$ is the treatment contrast. A convenient way to estimate $\mu _a({{\textbf{H}}})$ is to use the inverse-probability weighting (IPW) method, which leads to

5 $$\begin{aligned} {{\hat{C}}}^{\text{IPW}}({{\textbf{H}}}) ={\hat{\mu }}_1^{\text{IPW}}({{\textbf{H}}})-{\hat{\mu }}_0^{\text{IPW}}({{\textbf{H}}})= \left\{ \frac{A}{{\hat{\pi }}_1({{\textbf{H}}})} - \frac{1-A}{1-{\hat{\pi }}_1({{\textbf{H}}})} \right\} Y. \end{aligned}$$

Here, ${\hat{\pi }}_a({{\textbf{H}}}),~a\in \{0,1\}$ denotes the propensity score that can be estimated by imposing some parametric or nonparametric models given a set of covariates ${{\textbf{H}}}$. The IPW-based contrasting estimator in Eq. (5) is easily shown to be an unbiased estimator for $C({{\textbf{H}}})$, because of $E[I(A=a)/P(A=a|X=x)] =1$ and the consistency property of pseudo-observations. However, this approach is only valid when the propensity model $\pi _1({{\textbf{H}}})$ is correctly posited, which often fails to hold in practice, and usually it is statistically inefficient^35,36^. A more robust and efficient alternative is the augmented inverse-probability weighting (AIPW) estimator that combines outcome and propensity models to achieve the double-robustness property. Specifically, the AIPW estimator for $\mu _a$ takes the form

6 $$\begin{aligned} {\hat{\mu _a}}^{\text{DR}}({{\textbf{H}}}) = \dfrac{I(A=a)}{{\hat{\pi _a}}({{\textbf{H}}})} Y + \left\{ 1 - \dfrac{I(A=a)}{{\hat{\pi _a}}({{\textbf{H}}})} \right\} {\hat{\mu _a}}({{\textbf{H}}}), \end{aligned}$$

which is a weighted average between the pseudo-observation *Y* and its substitute ${\hat{\mu _a}}$ from an outcome regression model. Even if the target survival measure is non-negative, its pseudo-observation can take a positive or negative value^29^. Thus, it is natural to use a simple linear regression or modern machine learning techniques to approximate $\mu _a({{\textbf{H}}})$. In the statistical literature, (6) is well known as a double-robust (DR) estimator^10,20,37^, because it still produces a consistent result, when either the outcome model $\mu _a({{\textbf{H}}})$ (Q-model) or propensity score model $\pi _a({{\textbf{H}}})$ (A-model) is correctly imposed^37^.

In this work, we shall use (6) to obtain the DR contrast estimator, i.e., ${{\hat{C}}}^{\text{DR}}({{\textbf{H}}}) = {\hat{\mu }}^{\text{DR}}_{1}({{\textbf{H}}}) -{\hat{\mu }}^{\text{DR}}_{0}({{\textbf{H}}})$. Once this contrasting factor is computed, the optimal treatment regime $g^{\text{opt}}$ can be obtained from (4). However, weighted classification errors (4) may require complex and slow general algorithms because its optimization is not straightforward^9,13,34^. Zhang and Zhang ^13^ used a generic optimization algorithm via the *genoud* function from the *R: rgenoud* package. However, this function is computing expensive and works slowly when the covariate dimension is moderate-to-high. Instead, we propose to solve the classification problem (4) via the weighted linear SVM algorithm^38^, which can estimate the true treatment regime with high probability due to the Fisher consistency property^39^. Motivated by Song et al.^7^, we adopt a penalized SVM by incorporating the contrast function ${{\hat{C}}}^{\text{DR}}({{\textbf{H}}})$ as a weighting factor to achieve the optimization in (4). By letting $w_i=| {{\hat{C}}}_i^{\text{DR}}({{\textbf{H}}}_i) |$ and $Z_i = \text{sign}\{{{\hat{C}}}_i^{\text{DR}}({{\textbf{H}}}_i)\}$, the optimization problem in (4) may be accomplished by introducing a penalized hinge loss function and approximating (4) with

7 $$\begin{aligned} n^{-1}\sum _{i=1}^{n} w_i [1 - Z_i f({{\textbf{H}}}_i)]_+ + \sum _{j=1}^{p}P_\lambda (|\beta _j|), \end{aligned}$$

where $u_+ = \max (0,u)$ and $f(\cdot )$ is a prespecified function for treatment selection, so that $g^\text{opt}({{\textbf{H}}})=I\{f({{\textbf{H}}})>0\}$. For interpretability, we may take a simple linear decision function, i.e., $f({{\textbf{H}}}_i) = {{\textbf{H}}}_i^T{\varvec{\beta }},~{\varvec{\beta }}\in {\mathbb {R}}^p$. We also use the SCAD penalty function

$$\begin{aligned} P'_\lambda (|\beta _j|) = \lambda \left\{ I(|\beta _j| \le \lambda ) + \frac{(\gamma \lambda -|\beta _j|)_+}{\lambda (\gamma -1)}I(|\beta _j| > \lambda ) \right\} , \end{aligned}$$

where $\lambda > 0$ is a tuning parameter and $\gamma =3.7$ as recommended by Fan and Li^40^. Following a local linear approximation method, we further linearize the SCAD penalty term as

$$\begin{aligned} P_\lambda (|{\varvec{\beta }}| ) \approx P_\lambda (|{\varvec{\beta }}_0| ) + P_\lambda '(|{\varvec{\beta }}| )(|{\varvec{\beta }}| - |{\varvec{\beta }}_0|),~{\varvec{\beta }}\approx {\varvec{\beta }}_0, \end{aligned}$$

and introduce a slack variable $\xi _i = n^{-1} [1 - Z_if({{\textbf{H}}}_i)]_+$. Then the weighted classification problem in (7) can be recast as

8 $$\begin{aligned} &\underset{\xi _i,\beta _j^+,\beta _j^-, \beta _0^+,\beta _0^-}{\min } \sum _{i=1}^{n}w_i\xi _i + \sum _{j=1}^{p}P'_\lambda \left( |\beta _j^{(0)}|\right) \left( \beta _j^+ + \beta _j^-\right) \\ &\text{subject to } Z_i \left\{ \beta _0^+ - \beta _0^- + \sum _{j=1}^{p} h_{ij}\left( \beta _j^+ - \beta _j^-\right) \right\} \ge 1- \xi _i,\\ &\qquad \xi _i,\beta _0^+,\beta _0^-,\beta _j^+,\beta _j^-\ge 0,\quad \text{for}\quad i=1,\ldots ,n;\quad j=1,\ldots ,p, \end{aligned}$$

where $u^+ \ge 0$ and $u^-\ge 0$ are positive and negative parts of *u*, respectively, such that $u = u^+ - u^-$ and $|u| = u^+ + u^-$, and $h_{ij}$ is (*i*, *j*)th component of ${{\textbf{H}}}$. We may obtain an initial value $\beta _j^{(0)}$ from the standard $\ell _2$-type SVM optimization. There exist many optimization softwares to work on problem (8); for example, one may use the *lp()* function in the *R: lpSolve* package. After ${\hat{{\varvec{\beta }}}}$ is obtained, the estimated optimal treatment rule ${\hat{g}}^{\text{opt}}$ can be formulated as ${\hat{g}}^{\text{opt}}({{\textbf{H}}}) = I({{\textbf{H}}}^T{\hat{{\varvec{\beta }}}} > 0 )$. It is noted that lower *t*-year cumulative incidence rates are preferred under competing risks data. In this case, we can simply replace ${{\hat{C}}}_i^{\text{DR}}({{\textbf{H}}}_i)$ in (7) with $-{{\hat{C}}}_i^{\text{DR}}({{\textbf{H}}}_i)$ to minimize $F_{1}(t)$. This argument is justified by the following proposition.

#### **Proposition 1**

*The optimal treatment rule for competing risks outcome is the minimizer of the following weighted misclassification error*

$$\begin{aligned} g^{\textrm{opt}}( {{\textbf{H}}}) = {{\,{\hbox{arg min}}\,}}_{g \in {\mathscr{G}}}\,E_{{\textbf{H}}}[|C({{\textbf{H}}})| \{ I[C({{\textbf{H}}}) \le 0] \ne g({{\textbf{H}}}) \}]. \end{aligned}$$

### Dynamic treatment regimes

This section extends the previous argument to multi-stage treatment strategies to establish an optimal DTR. See Schulte et al.^10^ for more detailed description on this problem and related notations. To transfer the treatment effect between adjacent stages, we need to recursively define the value function at the stage-*k*^13^ as

9 $$\begin{aligned} V_k({{\textbf{H}}}_k) = E_{{\textbf{H}}}\left[ V_{k+1}({{\textbf{H}}}_{k+1})+ \eta _k\left\{ \mu _{1k}({{\textbf{H}}}_k)-\mu _{0k}({{\textbf{H}}}_k) \right\} \left\{ g^{\text{opt}}_k({{\textbf{H}}}_k)- A_k \right\} |{{\textbf{H}}}_k \right] , \end{aligned}$$

where $g^{\text{opt}}_k$ is the optimal treatment rule at *k*th stage and $\mu _{a_kk}({{\textbf{H}}}_k) = E_{{\textbf{H}}}[ V_{k+1}({{\textbf{H}}}_{k+1}) |A=a_k,{{\textbf{H}}}_k ]$ for $a_k\in \{0,1\}$. We set $V_{k+1} \equiv Y$ as there are no further subsequent processes. Note that $\mu _{a_k k}({{\textbf{H}}}_k)$ can be interpreted as a Q-function in reinforcement learning since it represents the “quality” of action $a_k$. Except for the last stage, $V_{k}({{\textbf{H}}}_{k})$ should be estimated backward in stages and let denote the estimated value function by ${\tilde{V}}_{k}\equiv {\tilde{V}}_{k}({{\textbf{H}}}_{k})$. The value function at the *k*th stage can be recursively estimated from the last stage by following equation ${\tilde{V}}_{k} = {\tilde{V}}_{k+1} + \eta _{k}\{ \hat{\mu }_{1k}({{\textbf{H}}}_{k})-{\hat{\mu }}_{0k}({{\textbf{H}}}_{k}) \} \{ {\hat{g}}^{\text{opt}}_k({{\textbf{H}}}_{k})- A_{k} \}$, where ${\tilde{V}}_{K+1} =Y$. Note that ${\tilde{V}}_{k}$ is equal to ${\tilde{V}}_{k+1}$ if the optimal treatment is given at the *k*th stage, i.e., ${\hat{g}}^{\text{opt}}_k({{\textbf{H}}}_{k}) = A_{k}$, otherwise $| \hat{\mu }_{1k}({{\textbf{H}}}_{k})-{\hat{\mu }}_{0k}({{\textbf{H}}}_{k}) |$ will be added to ${\tilde{V}}_{k+1}$. In the statistical literature, the appended term, which is equivalent to $| {\hat{\mu }}_{1k}({{\textbf{H}}}_{k})-\hat{\mu }_{0k}({{\textbf{H}}}_{k}) |I\{ {\hat{g}}^{\text{opt}}_k({{\textbf{H}}}_{k}) \ne A_{k}\}$, is called a “regret” function, because this quantity becomes positive when an optimal treatment is not given to the patient. The DTR algorithm aims to minimize this value at all stages of treatment regime to make it optimal. For the competing risks response, we should subtract the regret score from the $(k+1)$th value function to obtain the *k*th value function if the patient does not receive the optimal treatment, i.e., ${\tilde{V}}_{k} = {\tilde{V}}_{k+1} - \eta _{k}\{ {\hat{\mu }}_{1k}({{\textbf{H}}}_{k})-{\hat{\mu }}_{0k}({{\textbf{H}}}_{k}) \} \{ {\hat{g}}^{\text{opt}}_k({{\textbf{H}}}_{k})- A_{k} \}$, so that we could minimize the cause-specific risk in the end.

At each stage, we use parametric or nonparametric methods to obtain ${\hat{\mu }}_{a_kk}({{\textbf{H}}}_{k}),~a_k\in \{0,1\}$. The optimal treatment rule $g^{\text{opt}}_k\equiv g^{\text{opt}}_k({{\textbf{H}}}_k) =I( f({{\textbf{H}}}_{k}) > 0 )$ at the *k*th stage can then be determined by minimizing the expectation of the weighed misclassification error, $E_{{\textbf{H}}}[ \eta _k|C_k({{\textbf{H}}}_{k})|\{ I[ C_k({{\textbf{H}}}_{k})>0] \ne g_k({{\textbf{H}}}_{k}) \}]$. This can be done again by solving a $\ell _1$-type weighted linear SVM problem as in (8). Based on the value function ${\tilde{V}}_{k+1}$ from the $(k+1)$th stage, we can construct a DR estimator for the stage-*k* contrasting factor $C_k({{\textbf{H}}}_{k})=\mu _{1k}({{\textbf{H}}}_k)-\mu _{0k}({{\textbf{H}}}_k)$ as

10 $$\begin{aligned} {{\hat{C}}}_k^{\text{DR}} ({{\textbf{H}}}_{k}) = \frac{A_{k}\tilde{V}_{k+1}}{{\hat{\pi }}_1({{\textbf{H}}}_{k})} - \left\{ \frac{A_{k} - {\hat{\pi }}_1({{\textbf{H}}}_{k})}{{\hat{\pi }}_1({{\textbf{H}}}_{k})}\right\} \hat{\mu }_{1k}({{\textbf{H}}}_{k}) - \left[ \frac{(1-A_{k})\tilde{V}_{k+1}}{{\hat{\pi }}_0({{\textbf{H}}}_{k})} + \left\{ \frac{A_{k} - {\hat{\pi }}_1({{\textbf{H}}}_{k})}{{\hat{\pi }}_0({{\textbf{H}}}_{k})}\right\} \hat{\mu }_{0k}({{\textbf{H}}}_{k}) \right] , \end{aligned}$$

where ${\hat{\pi }}_{a_k}({{\textbf{H}}}_{k})$ is the estimated propensity score of $\pi _{a_k}({{\textbf{H}}}_{k})$. Notice that the estimated regret score in this case is equal to $|{{\hat{C}}}_k^{\text{DR}}({{\textbf{H}}}_k)|I\{{\hat{g}}^{\text{opt}}_k ({{\textbf{H}}}_k)\ne A_k\}$. Hence, the *k*th stage value function will be $\tilde{V}_k={\tilde{V}}_{k+1}+\eta _k |{{\hat{C}}}_k^{\text{DR}}({{\textbf{H}}}_k)|I\{{\hat{g}}^{\text{opt}}_k({{\textbf{H}}}_k)\ne A_k\}$. This computation proceeds in a backward iterative fashion from the last stage to the first, also related to dynamic programming algorithm^41^, which produces the desired optimal DTR, ${{{\textbf{g}}}}^\text{opt} = (g_1^\text{opt},\ldots ,g_K^\text{opt})$. We emphasize that the ${{{\textbf{g}}}}^\text{opt}$ may not be optimal unless the sequential randomization, consistency and positivity assumptions hold. Also, there may not be a unique ${{{\textbf{g}}}}^\text{opt}$. At any decision *k*, if there is more than one possible option $g_k^\text{opt}$ maximizing the potential reward outcome, then any rule $g_k^\text{opt}$ yielding one of these $a_k$ defines an optimal regime.

The proposed penalized DR-adjusted DTR estimation for survival outcome can be summarized as follows:

*Step 0.*: Set $${\tilde{V}}_{K+1} \equiv Y$$.
*Step 1.*: At stage-*k*, estimate $g^{\text{opt}}_k$ with $({{\textbf{H}}}_k,A_k,{\tilde{V}}_{k+1})$ by minimizing (7) with treatment contrast (10).
*Step 2.*: At stage-*k*, transfer the value function at stage-$(k+1)$ to the value function at stage-*k* with (9).
*Step 3.*: Set $k\leftarrow k-1$ and repeat steps 1 and 2 until $k=1$.

### Extension to DTR with multiple treatments

Thus far, it is assumed that the treatment option for $A_k$ is binary, i.e., $A_k=a_k\in \{0,1\}$. However, there are many clinical studies, testing more than two treatments, in which case the aforementioned approach for optimal treatment regime cannot be applied. With multiple treatment options, we will use a mixed approach of Huang et al.^8^ and Tao and Wang^12^. If there are $L_k\ge 3$ treatment options for the *k*th stage, we can consider the order statistics of $\mu _{a_k}({{\textbf{H}}}_k),~a_k=1,\ldots ,L_k,$, i.e., $\mu _{(1)}({{\textbf{H}}}_k)\le \cdots \le \mu _{(L_k)}({{\textbf{H}}}_k)$. Now let $\nu _{a_{k}}$ be the order index of the mean outcome, such that $\mu _{(a_k)}({{\textbf{H}}}_k) = \mu _{\nu _{a_{k}}}({{\textbf{H}}}_k)$. Then the best optimal treatment regime $g^{\text{opt}}_k$ among $L_k$ treatments may be estimated by directly maximizing

11 $$\begin{aligned} E_{{\textbf{H}}}\left[ \eta _k\sum _{a_k=1}^{L_k} \mu _{(a_k)}({{\textbf{H}}}_k) I\{\nu _{a_{k}}({{\textbf{H}}}_k) = g_k({{\textbf{H}}}_k) \} \right] . \end{aligned}$$

This optimization, however, is plausible only when $L_k$ is small and fixed in advance, otherwise it becomes very difficult to implement^8^. Alternatively, Tao and Wang^12^ suggested to find a sub-optimal treatment regime by paying attention to the following inequalities of the subsequent contrast functions for $a_k = 1,\ldots ,L_k-1$,

$$\begin{aligned} 0\le \mu _{(L_k)}({{\textbf{H}}}_k) -\mu _{(L_{k}-1)}({{\textbf{H}}}_k) \le \mu _{(L_k)}({{\textbf{H}}}_k) -\mu _{(a_k)}({{\textbf{H}}}_k) \le \mu _{(L_k)}({{\textbf{H}}}_k) -\mu _{(1)}({{\textbf{H}}}_k). \end{aligned}$$

By focusing on two specific contrasting factors $|\hat{\mu }_{(L_k)}({{\textbf{H}}}_k) - {\hat{\mu }}_{(L_k-1)}({{\textbf{H}}}_k) |$ and $| \hat{\mu }_{(L_k)}({{\textbf{H}}}_k) - {\hat{\mu }}_{(1)}({{\textbf{H}}}_k)|$ respectively, they identified sub-optimal treatment regimes as

12 $$\begin{aligned} {\hat{g}}^{\text{opt}}_k = \underset{g_k\in {\mathscr{G}}}{{{\,{\hbox{arg min}}\,}}}\, E_{{\textbf{H}}}[ \eta _k|\hat{\mu }_{(L_k)}({{\textbf{H}}}_k) - {\hat{\mu }}_{(L_k-1)}({{\textbf{H}}}_k) | I\{\nu _{L_{k}}({{\textbf{H}}}_k) \ne g_k({{\textbf{H}}}_k) \} ] \end{aligned}$$

and

13 $$\begin{aligned} {\hat{g}}^{\text{opt}}_k = \underset{g_k\in {\mathscr{G}}}{{{\,{\hbox{arg min}}\,}}}\, E_{{\textbf{H}}}[ \eta _k| \hat{\mu }_{(L_k)}({{\textbf{H}}}_k) - {\hat{\mu }}_{(1)}({{\textbf{H}}}_k)| I\{\nu _{L_{k}}({{\textbf{H}}}_k) \ne g_k({{\textbf{H}}}_k) \} ]. \end{aligned}$$

This argument suggests that a sub-optimal treatment rule may be obtained by controlling some of the treatment contrasting factors. Note that the decision rules in (12) and (13) minimize, respectively, the lower and the upper bounds of the expected loss in the outcome due to sub-optimal treatments in the entire population of interest. We explore both treatment selection methods in our numerical experiments with pseudo-observations for censored data. Our results reveal that the two methods produce similar performance. This may be because the minimum and maximum bounds of the objective function may converge to the same value unless the assumed models are severely mis-specified.

## Experimental studies

This section provides our empirical simulation results to demonstrate the finite-sample performance of the proposed method in a two-stage DTR setting. We also performed additional simulations, shown in the web-based supplementary material, which include the results for the single-stage estimation and covariate-dependent censoring situation.

### Scenario 1: Randomized experiments

We first evaluate the performance of the proposed method for the two-stage DTR problem when responses are subject to censoring and competing risks. Simulation results under single stage are postponed to the Tables S1 and S2 in the Web-appendix. We let $n=500$ or 1000 in all studies. Let $x_{k,ji}$ be the *j*th covariate value of the *i*th subject at the *k*th stage $( i=1,\ldots ,n; k = 1,2; j=1,\ldots ,p_k )$. At the first stage, we generate 10 covariates ${{\textbf{x}}}_{1,i} = (x_{1,1i}, \ldots ,x_{1,10i})^T$, where each covariate independently follows an Uniform$[-2,2]$ distribution. The second stage involves a single variable ${{\textbf{x}}}_{2,i} =(x_{2,i})$ that is generated from Uniform$[\min (x_{1,1i}), \max (x_{1,1i})]$. The treatment indicator $A_{k,i},~k=\{1,2\}$ is generated from Bernoulli(0.5). For survival outcome, we first generate first stage survival time as $T_{1,i} = \exp \{1.5+0.5x_{1,1i} + A_{1,i}(x_{1,2i}-0.5) + \epsilon _{1,i}\}$ and accumulated survival time at second stage as $T_{2,i} =\exp \{1.5+0.5x_{1,1i} + A_{1,i}(x_{1,2i}-0.5) + A_{2,i}(x_{2,i}-0.5)+ \epsilon _{2,i}\}$, where $\epsilon _{1,i}$ and $\epsilon _{2,i}$ are random error variables, independently generated from $\exp (\epsilon _{k,i})\sim \text{Exp}(1)$. Censoring times are generated from $C_i \sim \text{Exp}(c_0)$, where $c_0$ is a fixed constant yielding 15% or 30% censoring rates. A subject enters the second stage when $\eta _{2,i}=I(T_{1,i}<C_i)=1$. For an individual who is not alive at the beginning of the second stage (i.e., $\eta _{2,i}=0$), his or her survival time is $T_i=T_{1,i}\exp \{(g^{\text{opt}}_{2,i}- A_{2,i})(x_{2,i}-0.5)\}$, otherwise the survival time is given by $T_i=T_{2,i}$. That is, $T_i=\eta _{2,i}T_{2,i}+(1-\eta _{2,i}) T_{1,i}\exp \{(g^{\text{opt}}_{2,i}- A_{2,i})(x_{2,i}-0.5)\}$. In this setting, it can be shown that the optimal rules ${{{\textbf{g}}}}^\text{opt}=(g_1^\text{opt},g_2^\text{opt})$ are given by $g_1^\text{opt}= I(x_{1,2i} \ge 0.484)$ and $g_2^\text{opt}=I(x_{2,i}\ge 0.5)$. Under this setting, approximately 80% of individuals are transferred from stage 1 to stage 2. The propensity score for each individual is estimated by the sample proportion of the treatment, i.e., $\#(A_{k} = 1) / n$. Our objective is to find optimal DTRs that maximize the 3-year survival rate, for which the true maximal survival is known to be $S(3,{{{\textbf{g}}}}_0^\text{opt})=0.65$.

We further consider the competing risks data setting, in which we model the stage-1 and stage-2 Q-functions for the cause-1 event as $\psi _{1i} = \exp \{1 - 3x_{1,1i} - A_{1,i}(3.6x_{1,2i} -0.8)\}$ and $\psi _{1i} = \exp \{1 - 3x_{1,1i} - A_{1,i}(3.6x_{1,2i} -0.8) - A_{2,i}(0.5 - 1.7x_{2,i})\}$, respectively. The Q-model for the cause-2 event is specified as $\psi _{2i} = \exp \{1 + 3x_{1,1i} + A_{1,i}(x_{1,2i} + 0.8) - A_{2,i}(x_{2,i}-0.5)\}$. Following Fine and Gray^42^, we let $P_i(D_i=1) = 1 - (1 - q)^{1/\psi _{1i}}$ and generate the cause-2 event times from $F_{2i}(t) = 1 - \exp \{ -t\psi _{2i} \}.$ For the cause-1 event, we let $\eta _{2,i}=1$ if the cause-1 event time is less than 3. The cause-1 event times are generated from $F_{1i}(t) = 1 - \{ 1- q (1- e^{-t})\}^{\psi _{1i}}$ if $\eta _{2i}=1$, otherwise from $F_{1i}(t) = 1 - \{ 1- q (1- e^{-t})\}^{ \exp \{1 - 3x_{1,1i} - A_{1,i}(3.6x_{1,2i} -0.8)\} }$. With the choice of $q = 0.5$, about 43% and 38% of individuals experience the cause-1 failure, respectively, under 15% and 30% censoring rates. Also, approximately 45% are transferred to stage 2 and suffer from the cause-1 event. The optimal treatment rules are $g_1^\text{opt}= I(x_{1,2i} \le 0.250)$ and $g_2^\text{opt}= I(x_{2,i}\ge 0.294 )$, for which the true minimal 3-year cause-1 CIF is $F_1(3,{{{\textbf{g}}}}_0^\text{opt})=0.23$.

Table 1 summarizes the performance of several DTR methods, including outcome weighted learning (OWL)^39^ and its DR version (DWL), penalized OWL (POWL) and the proposed penalized DR weighted learning (PDWL), for survival and competing risks endpoints. In all cases, survival responses are replaced with their pseudo-observations. Here, OWL and POWL represents the pseudo-outcome weighted learning method and the SCAD-penalized OWL, respectively. For OWL and POWL, we evaluate the contrasting factor $C({{\textbf{H}}})$ by (5) and the value function by (9). Simulations are conducted to optimize the true survival curves, $\{S(3,{\hat{{{{\textbf{g}}}}}}^\text{opt}), F_1(3,\hat{{{\textbf{g}}}}^\text{opt}) \}$, and their empirical counterparts, $\{{{\hat{S}}}(3,{\hat{{{{\textbf{g}}}}}}^\text{opt}), {{\hat{F}}}_1(3,{\hat{{{{\textbf{g}}}}}}^\text{opt}) \}$. The results show that the proposed PDWL outperforms other algorithms, nearly achieving the maximal survival and minimal cumulative incidence rates in all cases. Our method also best performs in terms of correct decision rate at the first stage (CDR1) and average correct decision rate at both stages (ACDR), which are approximated with 50,000 test samples. Note that a naive treatment regime with ${{{\textbf{g}}}}=0$, i.e., just prescribing the control treatment in both stages, even produces better outputs than OWL or DWL. This implies that the performance of optimal treatment allocation rules can be greatly improved through penalization on the predictor-by-treatment interaction term.

**Table 1:** Performance of several DTR algorithms.

*n*	Censor	Method	Survival events				Cause-1 specific events			
			$S(3,{\hat{{{{\textbf{g}}}}}}^\text{opt})$	${{\hat{S}}}(3,{\hat{{{{\textbf{g}}}}}}^\text{opt})$	CDR1	ACDR	$F_1(3,{\hat{{{{\textbf{g}}}}}}^\text{opt})$	${{\hat{F}}}_1(3,{\hat{{{{\textbf{g}}}}}}^\text{opt})$	CDR1	ACDR
500	15%	${{{\textbf{g}}}}=0$	0.50 (0.00)	0.39 (0.02)	0.62 (0.00)	0.39 (0.00)	0.43 (0.00)	0.44 (0.02)	0.44 (0.00)	0.37 (0.00)
		${{{\textbf{g}}}}= 1$	0.31 (0.00)	0.39 (0.02)	0.38 (0.00)	0.14 (0.00)	0.42 (0.00)	0.43 (0.02)	0.56 (0.00)	0.14 (0.00)
		OWL	0.46 (0.05)	0.44 (0.05)	0.50 (0.10)	0.37 (0.10)	0.40 (0.04)	0.44 (0.04)	0.50 (0.09)	0.32 (0.10)
		DWL	0.50 (0.07)	0.50 (0.05)	0.85 (0.09)	0.47 (0.15)	0.28 (0.03)	0.33 (0.03)	0.85 (0.08)	0.54 (0.11)
		POWL	0.58 (0.02)	0.54 (0.04)	0.79 (0.08)	0.66 (0.08)	0.27 (0.02)	0.34 (0.03)	0.83 (0.06)	0.59 (0.09)
		PDWL	**0.61** (**0.01**)	**0.56** (**0.03**)	**0.90** (**0.04**)	**0.74** (**0.06**)	**0.26** (**0.01**)	**0.32** (**0.03**)	**0.89** (**0.03**)	**0.64** (**0.09**)
	30%	${{{\textbf{g}}}}= 0$	0.51 (0.00)	0.40 (0.02)	0.62 (0.00)	0.40 (0.00)	0.43 (0.00)	0.43 (0.02)	0.44 (0.00)	0.37 (0.00)
		${{{\textbf{g}}}}= 1$	0.32 (0.00)	0.40 (0.03)	0.38 (0.00)	0.14 (0.00)	0.42 (0.00)	0.43 (0.03)	0.56 (0.00)	0.14 (0.00)
		OWL	0.47 (0.05)	0.45 (0.05)	0.50 (0.10)	0.38 (0.10)	0.41 (0.04)	0.44 (0.04)	0.50 (0.09)	0.32 (0.10)
		DWL	0.50 (0.06)	0.50 (0.05)	0.84 (0.09)	0.44 (0.14)	0.28 (0.03)	0.33 (0.04)	0.85 (0.08)	0.53 (0.12)
		POWL	0.58 (0.03)	0.53 (0.04)	0.77 (0.09)	0.64 (0.08)	0.27 (0.02)	0.34 (0.03)	0.83 (0.07)	0.59 (0.09)
		PDWL	**0.61** (**0.01**)	**0.56** (**0.03**)	**0.89** (**0.04**)	**0.74** (**0.06**)	**0.26** (**0.01**)	**0.32** (**0.03**)	**0.89** (**0.03**)	**0.63** (**0.09**)
1000	15%	${{{\textbf{g}}}}= 0$	0.50 (0.00)	0.39 (0.01)	0.62 (0.00)	0.39 (0.00)	0.43 (0.00)	0.44 (0.01)	0.44 (0.00)	0.37 (0.00)
		${{{\textbf{g}}}}= 1$	0.31 (0.00)	0.39 (0.02)	0.38 (0.00)	0.14 (0.00)	0.42 (0.00)	0.43 (0.02)	0.56 (0.00)	0.14 (0.00)
		OWL	0.48 (0.05)	0.46 (0.05)	0.51 (0.11)	0.41 (0.11)	0.39 (0.05)	0.44 (0.04)	0.51 (0.10)	0.36 (0.11)
		DWL	0.51 (0.07)	0.52 (0.05)	0.90 (0.06)	0.51 (0.17)	0.27 (0.02)	0.32 (0.02)	0.88 (0.05)	0.59 (0.09)
		POWL	0.61 (0.01)	0.56 (0.03)	0.87 (0.06)	0.78 (0.07)	0.25 (0.01)	0.33 (0.02)	0.88 (0.05)	0.69 (0.08)
		PDWL	**0.62** (**0.01**)	**0.57** (**0.02**)	**0.93** (**0.02**)	**0.83** (**0.04**)	**0.25** (**0.01**)	**0.32** (**0.02**)	**0.91** (**0.02**)	**0.73** (**0.08**)
	30%	${{{\textbf{g}}}}= 0$	0.51 (0.00)	0.40 (0.02)	0.62 (0.00)	0.40 (0.00)	0.43 (0.00)	0.44 (0.02)	0.44 (0.00)	0.37 (0.00)
		${{{\textbf{g}}}}= 1$	0.32 (0.00)	0.40 (0.02)	0.38 (0.00)	0.14 (0.00)	0.42 (0.00)	0.43 (0.02)	0.56 (0.00)	0.14 (0.00)
		OWL	0.49 (0.05)	0.46 (0.05)	0.51 (0.11)	0.41 (0.12)	0.40 (0.05)	0.44 (0.04)	0.51 (0.10)	0.35 (0.12)
		DWL	0.51 (0.06)	0.52 (0.04)	0.89 (0.07)	0.46 (0.15)	0.27 (0.02)	0.32 (0.02)	0.88 (0.05)	0.59 (0.11)
		POWL	0.62 (0.02)	0.56 (0.03)	0.85 (0.07)	0.76 (0.07)	0.25 (0.01)	0.33 (0.02)	0.87 (0.05)	0.69 (0.08)
		PDWL	**0.63** (**0.01**)	**0.58** (**0.02**)	**0.93** (**0.02**)	**0.82** (**0.04**)	**0.25** (**0.01**)	**0.32** (**0.02**)	**0.91** (**0.02**)	**0.72** (**0.08**)

The table reports optimized *t*-year survival and *t*-year cumulative incidence rates, correct decision rate at first stage (CDR1), and average correct decision rate of two stages (ACDR). For each scenario, the best model is highlighted in bold.

### Scenario 2: Observational studies

We next consider observational studies, in which treatment selection is not randomized and may depend on patients’ histories. In a similar configuration to the first simulation, we consider two scenarios for the propensity score function: (i) true logistic: $P(A_{1,i} = 1|{{\textbf{x}}}_{1,i}) = \text{expit}( x_{1,2i} - 0.6 x_{1,3i} )$ and $P(A_{2,i} = 1|x_{2,i}) = \text{expit}(- 0.5x_{2,i} )$; and (ii) false logistic: $P(A_{1,i} = 1|{{\textbf{x}}}_{1,i}) = \text{expit}( x_{1,2i} - 0.6 x_{1,3i}-0.4 x_{1,3i}^2 )$ and $P(A_{2,i} = 1|x_{2,i}) = \text{expit}(- 0.5x_{2,i} - 0.2x_{2,i}^2 )$. Notice that the the true logistic models do not involve any second-order treatment effects, whereas the false logistic models have a quadratic term. We shall apply the standard logistic model with only main-effect terms, in which case the true logistic model is correctly specified but the false logistic model is mis-specified.

**Figure 1:**
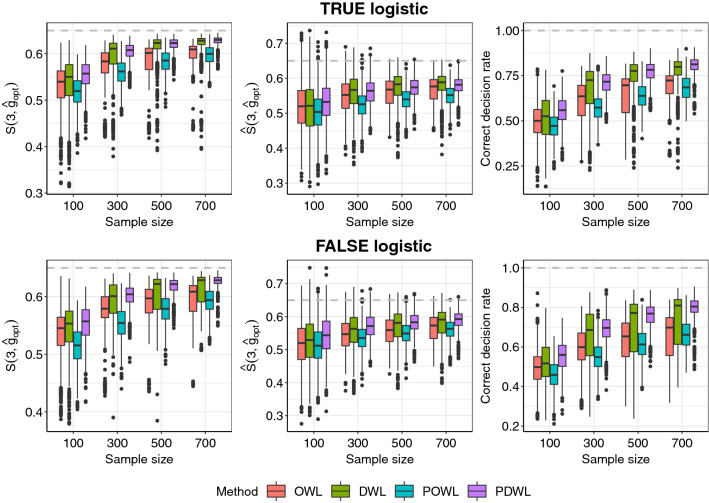
Survival probability and average correct decision rate (ACDR) of two stages under optimal dynamic treatment regimes with OWL, DWL, POWL and PDWL for different sample sizes. Optimal regimes should maximize the survival rate and ACDR.

**Figure 2:**
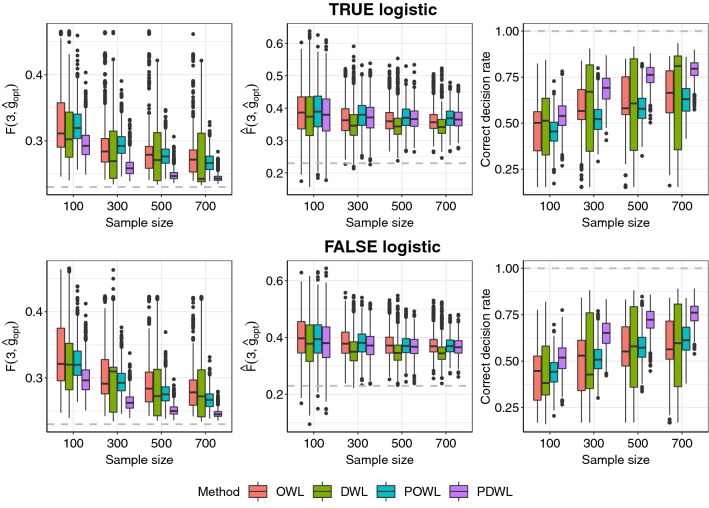
Cumulative incidence rate and average correct decision rate (ACDR) of two stages under optimal dynamic treatment regimes with OWL, DWL, POWL and PDWL for different sample sizes. Optimal regimes should minimize the cumulative incidence rate but maximize ACDR.

Figures 1 and 2 summarize the simulation results for the survival and competing risks endpoints, respectively, when censoring rates are about 30%. Again, four methods, OWL, DWL, POWL and PDWL, are compared in terms of the targeted survival measure and ACDR. Clearly, the proposed PDWL approach outperforms other algorithms, regardless of whether the fitted model is correctly specified or not, and also achieves the targeted optimal rates. Overall, DWL shows very high variability in predicting optimal regimes. On the other hand, POWL occasionally performs very poorly, even though its variation is well controlled. This implies that DR estimators should be accompanied with a proper penalization method to achieve optimal performance and that penalization alone could also result in inconsistent and misleading treatment rules. In almost all scenarios, OWL find sub-optimal rules and thus cannot be the method of choice. As the sample size increases, the performance of all algorithms improve.

### Scenario 3: Multiple treatments

**Figure 3:**
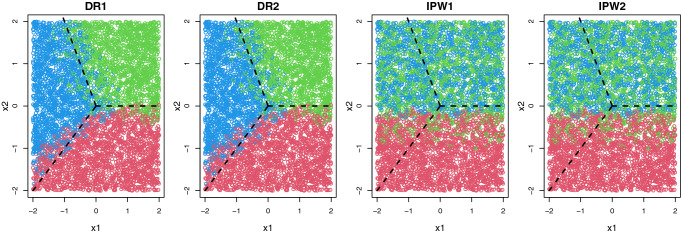
Treatment allocations of DR and IPW estimators when there are three treatment options, where the lower bound of contrast function (12) is applied to DR1 and IPW1 while the upper bound (13) is used in DR2 and IPW2, respectively.

Finally, we extend our method to the multiple treatments recommendation problem. For simplicity, we assume that there are three treatment options (i.e., $A_i\in \{1,2,3\}$) in a single-stage ($K=1$) setting. We let $x_{1i},x_{2i}$ and $x_{3i}$ follow Uniform[$-2,2$] independently and define $\varphi _{1i} = \exp (x_{2i} - 0.6x_{3i})$, $\varphi _{2i} = \exp (x_{2i} + 0.2x_{3i})$ and $\varphi _{3i} = 1+\varphi _{1i} + \varphi _{2i}$. Then, the treatment indicator $A_i$ is generated from a multinomial distribution with probabilities $(\varphi _{1i}/\varphi _{3i},\varphi _{2i}/\varphi _{3i},1/\varphi _{3i})$ for treatment 1, 2 and 3, respectively. The survival time is generated as $T_i = \exp \{ 1.5 + 0.5x_{1i} + (A_i =1) (x_{1i}-x_{2i}) + (A_i = 2)(x_{1i} + 0.5x_{2i}) + \epsilon _i \}$, where $\exp (\epsilon _i) \sim \text{Exp}(1)$. Figure 3 summarizes the results, where we use the one-versus-one SVM to optimize (11) under 30% censoring. Each color represents three treatments and black dashed line is the true decision line. Two DR methods (DR1 and DR2) perform well, clearly separating three treatment regions. In contrast, IPW-based methods (IPW1 and IPW2) result in poor classification performance, where treatment 1 is dominated by treatments 2 and 3. Here, DR1 and IPW1 are obtained from (12), whereas DR2 and IPW2 are based on (13). Clearly, doubly-robust modifications outperform basic estimators, which implies that model specification is also essential for the performance of classification algorithms.

## An application to ACTG175 data

### Data description

This section provides a practical application of the proposed treatment selection method to the AIDS Clinical Trial Group (ACTG175) study^43^. In this study, each subject was randomized by four treatment arms with equal assignment probabilities: (i) zidovudine monotherapy (ZDV), (ii) ZDV plus didanosine (ddI), (iii) ZDV plus zalcitabine (zal) and (iv) ddI monotherapy alone, which were coded as 0, 1, 2 and 3, respectively. Figure 1a visualizes the nonparametric survival curves for these four treatment arms, showing three treatment arms except ZDV alone have a similar survival rates. For this reason, previous work^24^ assumed that the treatment is binary by combining (ii)–(iv) into a single arm. In this analysis, we consider the optimal treatment selection problem between binary arms ((ii) versus (iii)) and among three treatment arms ((ii), (iii) and (iv)). The event of primary interest is the first observed time-to-event of either having a larger than 50% decline in the CD4 cell count or occurrence of immune deficiency syndrome or death. Twelve baseline covariates were considered in Hammer et al.^43^ and three of them were identified as important risk factors, which are age in year at baseline (Age), CD4 T-cell count at baseline (CD40) and Karnofsky score (Karnof). In addition to these three variables, we also include the following covariates in our analysis: Gender (Sex), weight in kilogram (Weight) and number of days of previously received antiretroviral therapy (Preanti). The overall censoring rate was 79.7% when the maximum follow-up time was set to 1000 days.

**Figure 4:**
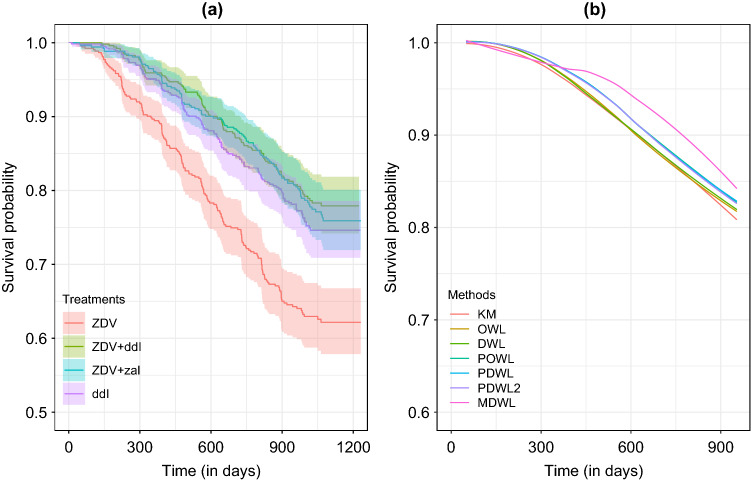
Nonparametric Kaplan–Meier curves of ACTG175 data under (**a**) given treatment arms and (**b**) optimal treatment rules.

### Analysis results

To examine whether the censoring distribution depends on a set of covariates, we first fitted a Cox proportional hazards model and we found that Sex and Preanti are statistically significant at the significance level of 0.05. Therefore, we considered modified pseudo-observations from Eq. (3) under the conditional independent censoring assumption as well as pseudo-observations from the standard Kaplan-Meier method. We computed individual pseudo responses for the survival rate after 1000 days since the treatment. Since this study was a randomized trial, we calculated the propensity score as the proportions of treated and untreated and applied a linear regression model to predict the mean response. Then we investigated seven methods for optimal treatment regime: (i) naive Kaplan–Meier, (ii) OWL, (iii) POWL, (iv) DWL, (v) PDWL, (vi) PDWL2, and (vii) MDWL. Here, PDWL2 represents the PDWL algorithm with covariate-adjusted pseudo-observations and MDWL represents the modified DWL algorithm for the three treatment options. The naive Kaplan–Meier curve under original treatment allocation is included as a reference.

Figure 4 shows (a) nonparametric Kaplan–Meier curves for four treatments and (b) the expected survival curves under the optimal treatment regimes from six weighted classification algorithms. Clearly, our proposed methods, PDWL and PDWL2, achieved higher overall survival probabilities than the other algorithms, although we focused on a particular *t*-year survival outcome. The performance of PDWL and PDWL2 were almost indistinguishable, implying that a covariate adjustment for the pseudo-value calculation may not make a noticeable difference in identifying optimal treatment regimes. Also note that OWL and DWL do not significantly improve the overall survival, compared to the naive KM estimator. This may show that penalization is critical in identifying an effective optimal treatment decision rule. The optimal survival rates, if patients followed the optimal treatment rules by PDWL and PDWL2 are above 83% at 1000 days after the treatment, whereas the survival rates under OWL and KM are less than 80% at the same time point. Finally, we note that the MDWL approach for multiple treatments can improve overall survival significantly, dominating the other methods after about 300 days. When implementing MDWL, two criteria (12) and (13) usually produce similar performance, and we used (12) to produce the result in Fig. 4b. This implies that although the suggested treatment rules for multiple treatments are sub-optimal, it could result in more improved performance than the two-treatment cases. More empirical and theoretical studies in this regard would be interesting.

## Discussion

In this paper, we propose an accountable survival contrast-learning to identify tailored optimal treatment regimes with time-to-event outcomes. Existing methodologies for censored data are mostly based on notoriously complex computing algorithms and become impracticable when the number of covariates are too much increased. It is partly because their procedures may involve a weighted nonparametric survival curve estimation at each iteration under potential population^24,25^. Alternatively, we employ an affordable pseudo-value approach by replacing unknown survival or competing risks measures with their jackknife-type resampling estimates. We then develop effective regularized survival contrast-learning algorithms that can produce interpretable optimal treatment rules. It should be also noted that many weighted classification algorithms are based on IPW estimating procedures with an $\ell _2$-penalization. However, these approaches are vulnerable to model mis-specification and amount of censoring and often underperformed as shown in our simulation studies. We provide empirical evidence that our proposal can significantly increase accountability and prediction power in tailoring clinical decision-making by combining well-known $\ell _1$-type regularization and doubly-robust weighting schemes. In real applications, however, linear treatment rules are sometimes not sufficient to achieve the maximum expected treatment reward and non-linear treatment rules may be requested. In that case, one may generalize the proposed SVM by using a reproducing kernel Hilbert space (RKHS) or pile multiple layers for the deep neural network (DNN). These architectures are widely used in many classification problems and can be explored under the DTR framework.

Of note, conventional pseudo-observations require the strict independent censoring condition, which may fail to hold in practice. Our empirical experiences, however, show that our approach still works well even in the case of covariate-dependent censoring. One may adopt an inversely censoring weighted approach to facilitate covariate-dependent censoring, as shown in Eq. (3)^30,31^, but we show that its contribution is limited in revealing optimal treatment rules. Further simulation results in Table S3 of the Web-appendix also show that the covariate-adjusted and unadjusted pseudo-value methods produce similar performance. Hager et al.^44^ also proposed an IPW-based classification algorithm for optimal dynamic treatment regime with censored survival data. Empirical studies to compare their algorithm with the proposed pseudo-value approach would be interesting. Finally, when there are multiple treatment arms, we used the sub-optimal contrast-learning classification algorithms that may not produce the globally optimal treatment rule. In this case, the classification algorithm may be applied several times to each pair among multiple treatment options. However, this approach is computationally demanding and also possibly subject to a multiple-testing problem. One might solve this problem by introducing SVM algorithms for multi-class items^45^. It is worth further investigation and will be pursued in a separate study.

## Supplementary Information

Supplementary Information.

## Data Availability

The pseudo-observation of survival quantities can be calculated by the R package pseudo^46^ and eventglm^32^. The optimization of the penalized SVM is conducted by the R package lpSolve^47^. One-versus-one pairwise SVM can be implemented by the R package e1071^48^. The ACTG175 dataset used in this study is available at the R package speff2trial^49^. The sample R code to implement our method is available via the first author’s Github (https://github.com/taehwa015/SurvDTR).

## References

[1] Murphy SA (2003). Optimal dynamic treatment regimes. J. R. Stat. Soc. Ser. B (Stat. Methodol.).

[2] Moodie EE, Richardson TS, Stephens DA (2007). Demystifying optimal dynamic treatment regimes. Biometrics.

[3] Zhao Y, Kosorok MR, Zeng D (2009). Reinforcement learning design for cancer clinical trials. Stat. Med..

[4] Qian M, Murphy SA (2011). Performance guarantees for individualized treatment rules. Ann. Stat..

[5] Tian L, Alizadeh AA, Gentles AJ, Tibshirani R (2014). A simple method for estimating interactions between a treatment and a large number of covariates. J. Am. Stat. Assoc..

[6] Chakraborty B, Murphy S, Strecher V (2010). Inference for non-regular parameters in optimal dynamic treatment regimes. Stat. Methods Med. Res..

[7] Song R (2015). On sparse representation for optimal individualized treatment selection with penalized outcome weighted learning. Stat.

[8] Huang X, Choi S, Wang L, Thall PF (2015). Optimization of multi-stage dynamic treatment regimes utilizing accumulated data. Stat. Med..

[9] Zhang B, Tsiatis AA, Laber EB, Davidian M (2012). A robust method for estimating optimal treatment regimes. Biometrics.

[10] Schulte PJ, Tsiatis AA, Laber EB, Davidian M (2014). Q- and A-learning methods for estimating optimal dynamic treatment regimes. Stat. Sci..

[11] Zhao Y-Q, Zeng D, Laber EB, Kosorok MR (2015). New statistical learning methods for estimating optimal dynamic treatment regimes. J. Am. Stat. Assoc..

[12] Tao Y, Wang L (2017). Adaptive contrast weighted learning for multi-stage multi-treatment decision-making. Biometrics.

[13] Zhang B, Zhang M (2018). C-learning: a new classification framework to estimate optimal dynamic treatment regimes. Biometrics.

[14] Qi Z, Liu Y (2018). D-learning to estimate optimal individual treatment rules. Electron. J. Stat..

[15] Lakkaraju, H. & Rudin, C. Learning cost-effective and interpretable treatment regimes. In *International Conference on Artificial Intelligence and Statistics* 166–175 (PMLR, 2017).

[16] Sherman, E., Arbour, D. & Shpitser, I. General identification of dynamic treatment regimes under interference. In *International Conference on Artificial Intelligence and Statistics* 3917–3927 (PMLR, 2020). PMC7730527 33313513

[17] Cai, H., Lu, W. & Song, R. On validation and planning of an optimal decision rule with application in healthcare studies. In *International Conference on Machine Learning* 1262–1270 (PMLR, 2020).

[18] Cui Y, Tchetgen Tchetgen E (2021). A semiparametric instrumental variable approach to optimal treatment regimes under endogeneity. J. Am. Stat. Assoc..

[19] Qiu H (2021). Optimal individualized decision rules using instrumental variable methods. J. Am. Stat. Assoc..

[20] Tsiatis, A. A., Davidian, M., Holloway, S. T. & Laber, E. B. *Dynamic Treatment Regimes: Statistical Methods for Precision Medicine* (Chapman and Hall/CRC, 2019).

[21] Simoneau G (2020). Estimating optimal dynamic treatment regimes with survival outcomes. J. Am. Stat. Assoc..

[22] Zhao Y-Q, Zhu R, Chen G, Zheng Y (2020). Constructing dynamic treatment regimes with shared parameters for censored data. Stat. Med..

[23] Zhao Y-Q (2015). Doubly robust learning for estimating individualized treatment with censored data. Biometrika.

[24] Jiang R, Lu W, Song R, Davidian M (2017). On estimation of optimal treatment regimes for maximizing $$t$$-year survival probability. J. R. Stat. Soc. Ser. B (Stat. Methodol.).

[25] Zhou J, Zhang J, Lu W, Li X (2021). On restricted optimal treatment regime estimation for competing risks data. Biostatistics.

[26] Robins, J. M. Optimal structural nested models for optimal sequential decisions. In *Proceedings of the Second Seattle Symposium in Biostatistics* 189–326 (Springer, 2004).

[27] Bai X, Tsiatis AA, Lu W, Song R (2017). Optimal treatment regimes for survival endpoints using a locally-efficient doubly-robust estimator from a classification perspective. Lifetime Data Anal..

[28] Andersen PK, Klein JP, Rosthøj S (2003). Generalised linear models for correlated pseudo-observations, with applications to multi-state models. Biometrika.

[29] Andersen PK, Pohar Perme M (2010). Pseudo-observations in survival analysis. Stat. Methods Med. Res..

[30] Binder N, Gerds TA, Andersen PK (2014). Pseudo-observations for competing risks with covariate dependent censoring. Lifetime Data Anal..

[31] Overgaard M, Parner ET, Pedersen J (2019). Pseudo-observations under covariate-dependent censoring. J. Stat. Plan. Inference.

[32] Sachs MC, Gabriel EE (2022). Event history regression with pseudo-observations: computational approaches and an implementation in R. J. Stat. Softw..

[33] Robins J (1986). A new approach to causal inference in mortality studies with a sustained exposure period-application to control of the healthy worker survivor effect. Math. Model..

[34] Zhang B, Tsiatis AA, Davidian M, Zhang M, Laber E (2012). Estimating optimal treatment regimes from a classification perspective. Stat.

[35] Lee BK, Lessler J, Stuart EA (2010). Improving propensity score weighting using machine learning. Stat. Med..

[36] McCaffrey DF (2013). A tutorial on propensity score estimation for multiple treatments using generalized boosted models. Stat. Med..

[37] Tsiatis A (2007). Semiparametric Theory and Missing Data.

[38] Cortes C, Vapnik V (1995). Support-vector networks. Mach. Learn..

[39] Zhao Y, Zeng D, Rush AJ, Kosorok MR (2012). Estimating individualized treatment rules using outcome weighted learning. J. Am. Stat. Assoc..

[40] Fan J, Li R (2001). Variable selection via nonconcave penalized likelihood and its oracle properties. J. Am. Stat. Assoc..

[41] Bather J (2000). Decision Theory: An Introduction to Dynamic Programming and Sequential Decisions.

[42] Fine JP, Gray RJ (1999). A proportional hazards model for the subdistribution of a competing risk. J. Am. Stat. Assoc..

[43] Hammer SM (1996). A trial comparing nucleoside monotherapy with combination therapy in HIV-infected adults with CD4 cell counts from 200 to 500 per cubic millimeter. N. Engl. J. Med..

[44] Hager R, Tsiatis AA, Davidian M (2018). Optimal two-stage dynamic treatment regimes from a classification perspective with censored survival data. Biometrics.

[45] Hsu C-W, Lin C-J (2002). A comparison of methods for multiclass support vector machines. IEEE Trans. Neural Netw..

[46] Perme, M. P. & Gerster, M. Pseudo: Computes pseudo-observations for modeling. R package version 1.4.3 (2017).

[47] Berkelaar, M., Eikland, K. & Notebaert, P. lpSolve: Interface to ‘Lp_solve’v. 5.5 to solve linear/integer programs. R package version 5.6.15 (2015).

[48] Meyer, D., Dimitriadou, E., Hornik, K., Weingessel, A. & Leisch, F. e1071: Misc functions of the Department of Statistics, Probability Theory Group (Formerly: E1071), TU Wien. R package version 1.7-4 (2020).

[49] Juraska, M. et al. speff2trial: Semiparametric efficient estimation for a two-sample treatment effect. R package version 1.0.4 (2012).

