# Supplementary material for: Accountable survival contrast-learning for optimal dynamic treatment regimes
Source: Sci Rep. 2023 Feb 8;13:2250. doi: 10.1038/s41598-023-29106-w (PMC9908913; doi:10.1038/s41598-023-29106-w)
Supplement: Supplementary file 1 — Supplementary Information. [file 41598_2023_29106_MOESM1_ESM.pdf]

The Web-based Supplementary Materials

for *"Accountable survival contrast-learning for optimal dynamic treatment regimes"*

by Taehwa Choi, Hyunjun Lee and Sangbum Choi

1 Proof of Proposition

This section provides the proof of the proposition by following the Zhang et al. [?]'s weighted classification scheme.

Proposition 1. *The optimal treatment rule for competing risks outcome is the minimizer of the following weighted misclassification error*

$$g^{opt}(\boldsymbol{H}) = \arg\min_{g\in\mathcal{G}} E_{\boldsymbol{H}}[|C(\boldsymbol{H})|\{I[C(\boldsymbol{H}) \leq 0] \neq g(\boldsymbol{H})\}].$$

Proof. Unlike other quantities, the cause-1 cumulative incidence function (CIF) should be minimized when a patient receives the optimal treatment. By using the unbiasedness property of pseudo-observation, it is observed that

$$\begin{aligned} F_1(t) &= E[\hat{F}_{1i}(t)] \\ &= E_{\mathbf{H}}[\mu_1(\mathbf{H})\{g(\mathbf{H}) = 1\} + \mu_0(\mathbf{H})\{g(\mathbf{H}) = 0\}] \\ &= E_{\mathbf{H}}[C(\mathbf{H})g(\mathbf{H}) + \mu_0(\mathbf{H})], \end{aligned}$$

where the second equation is followed by *consistency* assumption and $C(\mathbf{H}) = \mu_1(\mathbf{H}) - \mu_0(\mathbf{H})$. Note that the minimizer of the last equation about g is equivalent to the minimizer of $E_{\mathbf{H}}[C(\mathbf{H})g(\mathbf{H})]$. Then, by decomposing the contrast function $C(\mathbf{H})$ with its sign, we observe

$$E_{\mathbf{H}}[C(\mathbf{H})g(\mathbf{H})] = E_{\mathbf{H}}[I\{C(\mathbf{H}) > 0\}|C(\mathbf{H})|g(\mathbf{H}) - I\{C(\mathbf{H}) \leq 0\}|C(\mathbf{H})]$$

Furthermore, by adding and subtracting the term $I\{C(\mathbf{H}) > 0\}|C(\mathbf{H})|$, above equation can be expressed by

$$\begin{aligned} &E_{\mathbf{H}}[I\{C(\mathbf{H}) > 0\}|C(\mathbf{H})| - |C(\mathbf{H})|[\{1 - g(\mathbf{H})\}I\{C(\mathbf{H}) > 0\} + g(\mathbf{H})I\{C(\mathbf{H}) \leq 0\}]] \\ &= E_{\mathbf{H}}[I\{C(\mathbf{H}) > 0\}|C(\mathbf{H})| - |C(\mathbf{H})|[I\{C(\mathbf{H}) > 0\} - 2I\{C(\mathbf{H}) > 0\}g(\mathbf{H}) + g(\mathbf{H})]], \end{aligned}$$

where above equation can be simply verified by noting $I\{C(\mathbf{H}) > 0\} = 1 - I\{C(\mathbf{H}) \leq 0\}$, and the

last term of second equation can be expressed by quadratic term

$$F_1(t) = E_{\mathbf{H}}[I\{C(\mathbf{H}) > 0\}|C(\mathbf{H})|] - E_{\mathbf{H}}[|C(\mathbf{H})|[I\{C(\mathbf{H}) > 0\} - g(\mathbf{H})]^2].$$

Thus, minimization of $F_1(t)$ with respect to g is equivalent to maximizing $E_{\mathbf{H}}[|C(\mathbf{H})|[I\{C(\mathbf{H}) > 0\} - g(\mathbf{H})]^2]$,

$$\begin{aligned}
\arg\min_{g\in\mathcal{G}} F_1(t) &= \arg\min_{g\in\mathcal{G}} E[\hat{F}_{1i}(t)] \\
&= \arg\max_{g\in\mathcal{G}} E_{\mathbf{H}}[|C(\mathbf{H})|[I\{C(\mathbf{H}) > 0\} - g(\mathbf{H})]^2] \\
&= \arg\min_{g\in\mathcal{G}} E_{\mathbf{H}}[|C(\mathbf{H})|[I\{C(\mathbf{H}) \leq 0\} - g(\mathbf{H})]^2].
\end{aligned}$$

Finally, since the squared term in last equation consists only 1 or 0, we obtain the estimated optimal treatment regime by minimizing following weighted classification scheme under competing risks data

$$g^{\text{opt}}(\mathbf{H}) = \arg\min_{g\in\mathcal{G}} E_{\mathbf{H}}[|C(\mathbf{H})|I[I\{C(\mathbf{H}) \leq 0\} \neq g(\mathbf{H})]],$$

This implies that proposed method will generate the minimal value of the cause-1 CIF, which can be achieved by the true optimal treatment regime. □

2 Additional simulation results

We provide the results of numerical studies for single stage treatment allocation, under two different censoring assumptions. First, we consider completely independent censoring situation, i.e., $T \perp\!\!\!\perp C$. The censoring time C_i is generated from exponential distribution (i.e., $\text{Exp}(c_0)$), where the non-negative constant c_0 is fixed to yield approximately 15% or 30% censoring rate. Additionally, we fix our interest time point $t_0 = 3$ and 1000 Monte-Carlo simulation studies are conducted. We generate the 500 samples and 15 covariates $X_i = (x_{1i}, \ldots, x_{15i})^T$, which are independent and identically distributed with Uniform$[-2, 2]$. The treatment indicator $A_i \in \{0, 1\}$ is generated from binomial distribution with probability (i) $P(A_i = 1|x_i) = \text{expit}(x_{2i} - 0.6x_{3i})$ or (ii) $P(A_i = 1|x_i) = \text{expit}(x_{2i} + 0.6x_{2i}^2 - 0.6x_{3i})$, where $\text{expit}(a) = 1/(1 + \exp(-a))$. Since we posit the logistic model with x_{2i} and x_{3i}, the case (i) will be well specified model (True logistic) whereas the case (ii) will be wrong specified model (False logistic).

For the 3-year survival event, the survival time T_i is generated from $\exp\{1.5 + 0.5x_{1i} + A_i(x_{2i} - x_{3i}) + \epsilon_i\}$, where the error distribution ϵ_i follows extreme value distribution with rate 1 (i.e., $\exp(\epsilon_i) \sim \text{Exp}(1)$). True optimal treatment regime can be obtained from $g_0^{\text{opt}} = I(x_2 \geq x_3)$ which can be naturally derived to maximize the survival time T_i, and corresponding true maximal 3-year survival probability is $S(3, g_0^{\text{opt}}) = 0.63$. For the 3$-$year CIF in competing risks event, Suppose our

Table S1: Simulation results for optimal treatment regime with survival event under completely independent censoring.

		True logistic			False logistic		
Censor	Method	$S(3, \hat{g}^{\text{opt}})$	$\hat{S}(3, \hat{g}^{\text{opt}})$	CDR	$S(3, \hat{g}^{\text{opt}})$	$\hat{S}(3, \hat{g}^{\text{opt}})$	CDR
15%	$g = 0$	0.49 (0.00)	0.49 (0.02)	0.50 (0.00)	0.50 (0.00)	0.49 (0.02)	0.50 (0.00)
	$g = 1$	0.48 (0.00)	0.49 (0.03)	0.50 (0.00)	0.48 (0.00)	0.49 (0.02)	0.50 (0.00)
	OWL	0.59 (0.05)	0.60 (0.05)	0.78 (0.15)	0.57 (0.05)	0.57 (0.05)	0.73 (0.13)
	DWL	0.62 (0.02)	**0.62 (0.03)**	**0.88 (0.05)**	0.60 (0.03)	0.60 (0.03)	0.81 (0.08)
	POWL	0.60 (0.02)	0.59 (0.03)	0.78 (0.05)	0.60 (0.01)	0.60 (0.02)	0.81 (0.04)
	PDWL	**0.62 (0.01)**	0.61 (0.02)	0.87 (0.02)	**0.62 (0.01)**	**0.61 (0.02)**	**0.85 (0.02)**
30%	$g = 0$	0.50 (0.00)	0.49 (0.02)	0.50 (0.00)	0.50 (0.00)	0.49 (0.02)	0.50 (0.00)
	$g = 1$	0.48 (0.00)	0.49 (0.03)	0.50 (0.00)	0.48 (0.00)	0.49 (0.03)	0.50 (0.00)
	OWL	0.60 (0.04)	0.60 (0.04)	0.82 (0.10)	0.58 (0.04)	0.58 (0.04)	0.74 (0.11)
	DWL	**0.62 (0.02)**	**0.62 (0.03)**	**0.88 (0.05)**	0.60 (0.03)	0.60 (0.03)	0.81 (0.08)
	POWL	0.59 (0.02)	0.59 (0.03)	0.77 (0.05)	0.60 (0.01)	0.60 (0.02)	0.81 (0.04)
	PDWL	**0.62 (0.01)**	0.61 (0.02)	0.87 (0.02)	**0.62 (0.01)**	**0.61 (0.02)**	**0.85 (0.02)**

model is generated from the subdistribution hazard model [?] with two competing risks ($D = 1, 2$). Let $\psi_{1i}(X) = \exp\{-1+1.2x_{1i}+A_i(x_{2i}-x_{3i})\}$ and $\psi_{2i}(X) = e^{-1-A(2+x_{1i}+2x_{2i})}$ be the true Q-function of cause-1 and 2 events, respectively. We generate the cause-1 event from binomial distribution with probability $P(D_i = 1) = 1 - (1-q)^{\exp\{-1+1.2x_{1i}+A_i(x_{2i}-x_{3i})\}}$. Here $q \in (0, 1]$ is a fixed constant controlling the proportion of cause-1 which produces roughly 54% for 15% censoring and 48% for 30% censoring of the data with $q = 0.8$ in this case. Under this consideration, with $P(D_i = 1)$, cause-1 failure time is generated from $F_1(t|X_i, A_i) = 1-\{1-q(1-e^{-t})\}^{\psi_{1i}(X)}$ whereas cause-2 failure time is generated as follows with probability $1-P(D_i = 2)$, $F_2(t|X_i, A_i) = 1-\exp\{-t\psi_{2i}(X)\}$. True optimal treatment regime for minimizing cause-1 CIF is $g_0^{\text{opt}} = I(x_2 \leq x_3)$, and the corresponding minimal cause-1 CIF is $F_1(3, g_0^{\text{opt}}) = 0.36$.

Table S1 and S2 are the results of 3-year survival probability and cause-1 CIF, respectively. Equivalent to the main manuscript, we consider two naive approaches ($g = 0$ and $g = 1$) and four models: outcome weighted learning (OWL[?]) and its DR version (DWL), penalized OWL (POWL) and the proposed penalized DR weighted learning (PDWL). Each model is compared by using four criteria, true curves $\{S(3, \hat{g}^{\text{opt}}), F_1(3, \hat{g}^{\text{opt}})\}$ and its empirical counterparts $\{\hat{S}(3, \hat{g}^{\text{opt}}), \hat{F}_1(3, \hat{g}^{\text{opt}})\}$, and correct decision rate with 50,000 new test data. For each scenario, the best model is highlighted in bold. The empirical standard errors are presented in parentheses. In general, DWL and PDWL show satisfactory performances, while the PDWL show more stable results under increased censoring rate. We can further see that the PDWL has minimal standard error in general case.

Next, we consider the covariate-dependent censoring situation. The censoring time is generated from $C_i \sim \text{Exp}(-c_0 - x_1 - x_2)$, where c_0 is a constant yielding target censoring proportion. Other set-ups are equal to previous ones. Given the true optimal treatment regime $g_0^{\text{opt}} = I(x_2 \leq x_3)$, the maximal 3-year survival probability $S(3, g_0^{\text{opt}})$ is given by 0.65. The results of simulations

Table S2: Simulation results for optimal treatment regime with competing risks event under completely independent censoring.

		True logistic			False logistic		
Censor	Method	$F_1(3,\hat{g}^{\text{opt}})$	$\hat{F}_1(3,\hat{g}^{\text{opt}})$	CDR	$F_1(3,\hat{g}^{\text{opt}})$	$\hat{F}_1(3,\hat{g}^{\text{opt}})$	CDR
15%	$g=0$	0.48 (0.00)	0.47 (0.02)	0.50 (0.00)	0.48 (0.00)	0.48 (0.02)	0.50 (0.00)
	$g=1$	0.48 (0.00)	0.48 (0.03)	0.50 (0.00)	0.48 (0.00)	0.48 (0.03)	0.50 (0.00)
	OWL	0.40 (0.04)	0.37 (0.05)	0.79 (0.15)	0.42 (0.04)	0.41 (0.05)	0.70 (0.14)
	DWL	**0.37 (0.01)**	**0.34 (0.03)**	**0.88 (0.03)**	**0.38 (0.02)**	0.38 (0.03)	0.81 (0.08)
	POWL	0.40 (0.01)	0.38 (0.03)	0.74 (0.06)	0.39 (0.01)	0.39 (0.03)	0.77 (0.05)
	PDWL	0.38 (0.00)	0.35 (0.03)	0.85 (0.03)	**0.38 (0.01)**	**0.37 (0.03)**	**0.82 (0.03)**
30%	$g=0$	0.48 (0.00)	0.47 (0.03)	0.50 (0.00)	0.48 (0.00)	0.48 (0.03)	0.50 (0.00)
	$g=1$	0.48 (0.00)	0.47 (0.03)	0.50 (0.00)	0.48 (0.00)	0.48 (0.03)	0.50 (0.00)
	OWL	0.39 (0.03)	0.37 (0.04)	0.82 (0.11)	0.41 (0.03)	0.41 (0.04)	0.74 (0.11)
	DWL	**0.38 (0.01)**	**0.36 (0.03)**	**0.87 (0.05)**	0.39 (0.02)	**0.39 (0.04)**	0.80 (0.07)
	POWL	0.41 (0.02)	0.40 (0.04)	0.72 (0.06)	0.40 (0.01)	0.40 (0.03)	0.76 (0.05)
	PDWL	**0.38 (0.00)**	**0.36 (0.03)**	0.85 (0.03)	**0.38 (0.00)**	**0.39 (0.03)**	**0.82 (0.03)**

Table S3: Simulation results for optimal treatment regime with survival event under covariate-dependent censoring.

		True logistic			False logistic		
Censor	Method	$S(3,\hat{g}^{\text{opt}})$	$\hat{S}(3,\hat{g}^{\text{opt}})$	CDR	$S(3,\hat{g}^{\text{opt}})$	$\hat{S}(3,\hat{g}^{\text{opt}})$	CDR
15%	$g=0$	0.51 (0.00)	0.51 (0.02)	0.50 (0.00)	0.51 (0.00)	0.51 (0.02)	0.5 (0.00)
	$g=1$	0.49 (0.00)	0.51 (0.03)	0.50 (0.00)	0.49 (0.00)	0.51 (0.03)	0.5 (0.00)
	OWL	0.62 (0.04)	0.61 (0.04)	0.82 (0.10)	0.59 (0.05)	0.59 (0.04)	0.74 (0.11)
	DWL	**0.64 (0.02)**	**0.63 (0.03)**	**0.89 (0.05)**	0.61 (0.03)	0.61 (0.04)	0.78 (0.08)
	POWL	0.61 (0.02)	0.61 (0.02)	0.78 (0.05)	0.62 (0.01)	0.61 (0.02)	0.81 (0.04)
	PDWL	**0.64 (0.01)**	**0.63 (0.02)**	0.87 (0.02)	**0.63 (0.01)**	**0.62 (0.02)**	**0.85 (0.02)**
	PDWL2	**0.64 (0.01)**	0.61 (0.02)	0.86 (0.02)	**0.63 (0.01)**	0.61 (0.02)	**0.85 (0.02)**
30%	$g=0$	0.52 (0.00)	0.53 (0.02)	0.50 (0.00)	0.52 (0.00)	0.52 (0.02)	0.50 (0.00)
	$g=1$	0.51 (0.00)	0.53 (0.03)	0.50 (0.00)	0.51 (0.00)	0.52 (0.03)	0.50 (0.00)
	OWL	0.65 (0.03)	**0.64 (0.03)**	0.84 (0.07)	0.62 (0.04)	0.62 (0.04)	0.76 (0.09)
	DWL	**0.66 (0.02)**	**0.64 (0.03)**	**0.88 (0.05)**	0.63 (0.03)	0.63 (0.03)	0.79 (0.08)
	POWL	0.63 (0.02)	0.62 (0.03)	0.77 (0.05)	0.64 (0.01)	0.63 (0.02)	0.80 (0.04)
	PDWL	**0.66 (0.01)**	**0.64 (0.02)**	0.87 (0.02)	**0.65 (0.01)**	**0.64 (0.02)**	**0.85 (0.02)**
	PDWL2	0.65 (0.01)	0.61 (0.03)	0.86 (0.02)	**0.65 (0.01)**	0.60 (0.03)	**0.85 (0.03)**

are summarized in Table S3. Here, we further simulate "PDWL2", where pseudo-observation is adjusted by inverse-probability of censoring weights with `R:eventglm` package [**?**]. Proposed methods still show satisfactory results not being biased although we assume covariate-dependent censoring, and there are no noticeable differences among double-robust methods between covariate-adjusted and covariate-unadjusted approaches.
